# Dyadic Interactions, Communication and Regulation Skills: Associations with Screen Use in Toddlers from Buenos Aires

**DOI:** 10.11621/pir.2024.0403

**Published:** 2024-12-01

**Authors:** Lucas G. Gago-Galvagno, María del Pilar Castillo, Marcos A. Fernández, Angel J. Tabullo, Stephanie E. Miller, Angel M. Elgier, Susana C. Azzollini

**Affiliations:** a Interamerican Open University, Argentina; b University of Buenos Aires, Argentina; c Institute of Human, Social and Environmental Sciences (INCIHUSA), Argentina; d National Council for Scientific and Technical Research (CONICET), Argentina; e University of Mississippi, Oxford, USA

**Keywords:** toys, screen, parent–child interaction, regulation, communication, toddlers

## Abstract

**Background:**

Screen time has increased, with more frequent use at younger ages during the developmental process. International pediatric associations recommend that its use be minimal before three years of age. However, several studies have shown that in this age range, its use is for at least one hour per day, and in general without the accompaniment of an adult and with no consideration of age-appropriate content. Furthermore, negative associations between screen use in hours and minutes were reported with different cognitive abilities (e.g., language, executive functions, attention, memory) during this period. Many of the studies carried out on associations between these variables used questionnaires or parental reports. This is why it becomes important to study how screen time is associated with early interactions between primary caregiver and toddlers and with early cognitive skills, using measures that observe behavior directly, and in a non-WEIRD sample [a WEIRD population is White, Educated, Industrialized, Rich, and Democratic – ed.] from low-to-medium SES backgrounds in Latin America. This could generate interventions to promote early cognitive development, and evaluate what type of responsible use can be provided for screen consumption in the early years.

**Objective:**

To describe the use of screens in toddlers of low-to-medium SES, compare caregiver–toddler interactions when engaged in play with digital or physical stimuli (with screens or toys), and examine screen use associations with regulation, early communication skills, and sociodemographic variables.

**Design:**

A mixed quantitative research sample was of 33 dyads of low-to-medium-SES primary caregivers and toddlers from 12 to 36 months (M.age = 27.2 months, SD = 7.04, female = 16) from Buenos Aires, Argentina. Sociodemographic and screen use questionnaires, cognitive tasks of regulation and communication, and two free-play sessions of six minutes (i.e., with toys and screens) were used.

**Results:**

Caregivers reported that their toddlers were exposed to TV, background TV, and cell phones for more than one hour per day with different content types. Caregivers generally preferred toys to screens, had a negative view of screens, and reported using them to distract their toddlers. Play sessions with toys promoted more verbal and non-verbal interactions between caregivers and toddlers, and these interactions were positively related to cognition. Also, TV use had differential correlations with toddlers’ interactions depending on whether it involved verbal or non-verbal communication. Finally, negative associations of TV and background TV with cognitive and socioeconomic variables were found.

**Conclusion:**

It would be important to encourage participation in traditional games or other face-to-face interaction activities and develop interventions focused on parent education-related screen use, child development, and tips for engaging in quality interactions with toddlers.

## Introduction

The American Association of Pediatrics (APP) produced a guide for parents in 1999 (revised in 2011 and 2013) that focused on prohibiting screen time in toddlers and restricting it to two hours per day for older children. Before publishing its revised findings and recommendations in 2016 (Council on Communications and Media, 2016a; 2016b), the AAP conducted a review of up-to date-evidence (Chassiakos et al., 2016). The new guidelines established that toddlers should not be exposed to screens until they are 18 months old unless these are interactive media such as video calls. Starting at 18 months, educational content is recommended if caregivers participate and interact. From 2 to 5 years old, the caregiver should help interpret the content, and usage time should be restricted to one hour per day. In any case, today the APP (APP, n.d.) suggests that, since children and adolescents can have many different types of interactions with technology, it is recommended to consider the quality of interactions with digital media instead of establishing specific time restriction guidelines. Other entities (Canadian Pediatric Society, 2017) also recommend that screen use be supervised by an adult to prioritize educational content appropriate for the age, and that does not interfere with sleep or communication.

Several papers have reported that the use of screens has increased in the daily lives of toddlers (Chen & Adler, 2019; Grané, 2021; Madigan et al., 2020; Rayce et al., 2024; Sas & Estrada, 2021; Simaes et al., 2022). Bergmann et al. (2022) published results regarding the screen use of 2,209 infants and toddlers aged 8–36 months during pandemic quarantines in 12 countries. Caregivers reported that even without online education requirements, toddlers were exposed to more screen time during lockdown than before it. Along the same lines, a meta-analysis found that only a quarter of children under 2 years old and a third between 2 and 5 years old meet the screen time guidelines. In addition, those who use screens 2 hours or more a day are more likely to have behavioral problems and lower cognitive skill scores (McArthur et al., 2022).

Among the negative effects of screen use are increased adiposity; sleep problems and aggressive behaviors; worse scores in executive functions and motor development; fewer physical activities and more sedentary activities, along with worse behavioral and emotional outcomes (Li et al., 2020). Furthermore, recent lines of evidence suggest a negative impact of screen exposure on children’s brain development. Structural magnetic resonance imaging (MRI) showed that screen use affected the microstructural integrity of the brain’s white matter in preschool children (Hutton et al., 2020). A further study found that a composite measure of screen times predicted lower cortical thickness and sulcus depth in children’s temporal, parietal, and occipital cortexes, indicating a potentially detrimental effect of screen exposure on brain development (Hutton et al., 2022). Along the same lines, a recent longitudinal study showed that children’s longer daily screen times were associated with lower connectivity within fronto-striatal circuits involved in inhibitory control, and this effect was mediated by an increased sensitivity to short-term rewards (Chen et al., 2023).

Regulation is an essential capacity for cognitive development, since it predicts language, social skills, mental health, academic achievement, and the type of occupation in later years (Ahmed et al., 2019; Moffitt et al., 2011; Woodward et al., 2017). Based on parental reports and behavioral measures, excessive exposure to TV and cell phones at home generates a decrease in regulatory abilities in preschool children (Munzer et al., 2019; Nathanson et al., 2014; Radesky et al., 2014). Media exposure (computer, cell phone, TV) in the first years of life negatively predicts subsequent regulatory capacities (Cliffet al., 2018; Lee et al., 2024). Lee et al. (2024) found in a meta-analysis that there was a significant association between the frequency of screen use by children and performance of regulatory tasks: the longer the toddlers used them, the lower their regulatory performance.

Regarding language, the meta-analysis by Madigan et al. (2021) associated more screen time and background TV with smaller child vocabularies. However, better quality of screen use (educational and co-viewing) was found to be positively associated with a child’s language skills. In another integrative review study (Gago Galvagno et al., 2022b), it was found that these technological devices were negatively associated with communication skills, although this was moderated by the company and scaffolding of caregivers during the activity. Rayce et al. (2024) found in a sample of 31,125 Danish children that excessive screen use (> 1 hour) was negatively associated with the language of toddlers aged 2 to 3 years. Empirical studies have reached similar results (Medawar et al., 2023; Panjeti-Madan et al., 2023). Longitudinal studies showed lower cognitive abilities during preschool years if the age of onset was during toddlerhood, but no long-term effects were studied (McArthur et al., 2020; Supanitayanon et al., 2020). Most of the studies included in these reviews used parent reports, both for screen use and cognitive abilities (Gago Galvagno et al., 2022b, Madigan et al., 2021). Although most of these international studies demonstrate negative relationships between screen use, cognition, and language, it is necessary to highlight that these studies do not show negative relationships between all measures of screen use, cognition, and language development (e.g., some have contradictory results or lack significance). Other studies find no negative associations between screen use, cognitive development, and language (Karani et al., 2022; Li et al., 2020). For example, two other meta-analyses on the relationship between screen time and psychological impacts (Ophir et al., 2021) and executive functions (EF) (Bustamante et al., 2023) found no significant statistical associations between the key variables. Some studies demonstrating benefits (e.g., problem-solving, imitation ability and word learning, improvements in mathematics and reading ability of preschool children) are found when considering variables such as context, content, or the type of interaction that took place between the toddler/child and the caregiver (Madigan et al., 2021; Medawar et al., 2022; Xie et al., 2018). Thus, results in this area are mixed.

One common hypothesis for why screen use may show negative effects on cognition and language is the displacement theory. This suggests that screen use may come at the expense of time engaged with more developmentally appropriate and rich stimuli important for development (e.g., involving quality reciprocal human interaction and exploration in the physical environment, Bustamante et al., 2023). Research examining children’s interactions with physical toys aligns with this hypothesis, as toy interaction has been shown to be important for cognitive development since it stimulates symbolic games, problem-solving, physical activity, self-regulation, and social and linguistic interactions (Milteer et al., 2012; Quinn et al., 2018). Furthermore, several researchers have found that human interaction such as child-directed language and caregiver support for the activity is positively associated with children’s cognitive development, regardless of the mediating object they are sharing (Bukhalenkova et al., 2023; Duch et al., 2013; Foursha-Stevenson et al., 2017; Medawar et al., 2022). However, it has been seen that play experiences in early childhood are compromised by the impoverished quality of interactions with primary caregivers during joint play with electronic games (battery-operated or digital) or mediated through screens (Carr & Dempster, 2021; Munzer et al., 2019). Lee and Wood (2021) examined support and scaffolding in 32 dyads using physical and virtual blocks and puzzles in 10-minute sessions. In general, caregivers provided more support in the 3D context than in 2D. On the other hand, Archer et al. (2021) observed 30 dyads (with children aged 12 to 24 months) as they introduced and interacted with novel and familiar mobile technologies and found that familiarity with the device was associated with fewer scaffolds, interactions, and more passive activities. Second, more varied verbal scaffolding was related to higher developmental scores, although when faced with new technologies, parents showed more verbalizations with older children. Finally, the researchers propose that children’s interest in mobile technology is not inherent and increases with age.

These results could be interpreted considering Sociocultural Psychology theory. A mediating object is any tool, symbol or artifact that facilitates the interaction between the individual and his or her social environment, allowing the co-construction of knowledge. Screens could be objects that do not include interactions with others and require mostly passive consumption, while traditional toys, such as building blocks, dolls or puzzles, act as mediating objects that invite the active participation of both the child and the caregiver. Through these toys, caregivers can guide toddlers in their learning, in what L.S. Vygotsky (1978) called the zone of proximal development (ZPD), where the child is able to perform tasks with the help of another, but not yet independently (Archer et al., 2021).

Finally, it has been found that social vulnerability is associated with greater use of screens and less time sharing with toddlers (Celik et al., 2021; Gago-Galvagno et al., 2023). In the last third of 2022, statistical evidence shows that, in Latin America, the poverty rate was 32.1% and extreme poverty 13.1% (CEPAL, 2022). At the beginning of 2024, there were high levels of social inequality and inflation in most of the countries of the region, with a Gini index greater than .40 (World Bank, 2024). It should be noted that socioeconomic status (SES) was negatively associated with screen time during confinement and positively with caregiver screen time, caregiver attitudes toward toddler screen time, and the age of the child. These features make it important to conduct research in these samples from disadvantaged social contexts to better understand the differences in screen use and its relationship to language and cognition, which will be important to developing effective interventions tailored to toddlers’ contextual backgrounds.

The gaps in scientific knowledge the present research aims to fill are due to the lack of studies that use cognitive and behavioral tasks instead of relying solely on questionnaires or parental reports in samples of toddlers from low- to middle-income countries, specifically in Buenos Aires, Argentina. Much of the previous research on screen use and interactions with caregivers has focused on preschool children in high-income countries (WEIRD samples) and has used methods that may be more susceptible to bias, such as parental reports. Another goal of this study is to continue finding results, given that previous studies have shown mixed outcomes regarding the impact of screens on cognition in this age range.

The research questions of this study are: Is there a difference on observational dyadic verbal and non-verbal interactions between caregivers and toddlers aged 12 to 36 months from low-to-medium SES from Buenos Aires, depending on which mediating object (screen or standardized toys) they used for 5-minute free-play sessions? How are parents’ reports of toddler’s screen time use with different devices (TV, PC, Cell Phone and Tablet) related to communication (observational dyadic interactions and expressive and receptive abilities) and regulation skills (working memory, cognitive flexibility, and inhibition), considering sociodemographic variables in a non-WEIRD sample? To respond these questions, the objectives of the research are to describe the caregivers’ reports of time use and the content of toddlers’ screen usage; explore caregivers’ perceptions of screens use among toddlers; compare the interactions of dyads in free-play sessions with traditional toys and screens; and associate communication and regulation skills with screen time, sociodemographic variables, and interactions during free-play sessions. Based on our review of the literature and Sociocultural Psychology, the hypotheses of this study are a) screen times in 1-to-3-year-old toddlers from low-to-medium-SES will exceed those recommended by pediatric associations and with diverse types of content; b) more verbal and non-verbal interactions will be observed in free-play sessions with toys compared to screen-based sessions; c) higher screen use will be negatively correlated with cognitive skills and SES.

## Methods

### Participants

Sampling was non-probabilistic, intentional, and snowball type. From 36 dyads, 4 children were excluded because of age (>36 months) and 2 because of prematurity. We thus evaluated 33 caregivers and toddlers (M.age = 27.2 months, SD = 7.04, female = 16, range 12–36 months) from low-to-medium-SES attending daycare centers (n = 28) and at homes in shantytowns of the Autonomous City and the Province of Buenos Aires (n = 4). The daycare centers were accessed through a regional director of the institutes, which also provided contacts from caregivers who were interested in participating in their homes.

### Measures

*Ad-hoc Sociodemographic Questionnaire:* Data were collected on gender, age, nationality, and city where the toddler resides. Questions were asked about the relationship of the interviewee to the toddler, the toddler’s health history, and the educational level and occupation of the caregiver. Socioeconomic questions were asked such as: how many people live in the home, the number of bedrooms in the home, whether there is a bathroom at home, and whether in the last six months, the household income co vered basic needs related to food and healthcare. A composite score of the amount of unmet basic needs (UBN) was formed considering overcrowding, caregivers’ educational level (incomplete secondary or less), occupation (or unemployed ), la ck of a bathroom in the home, less than 3 or 4 meals a day, and lack of access to healthcare.

*Screen Use:* We asked how many hours, in a typical day, the toddler was exposed to background TV, TV, PC, cell phones, and tablets; the type of content that was predominantly consumed (entertainment, music, educational) and if it was appropriate for the age (for adults, for toddlers, both). In addition, the caregiver was asked open questions about whether it is better to use screens or traditional toys with their toddlers, whether they knew the recommendations regarding the responsible use of screens from the national and international Pediatric Societies, and why they leftthe toddler alone to use various screens.

*Free Play with Toys and Screens:* To explore the interaction behaviors during the sessions with the use of cell phones and toys, the free-play procedure was applied that is widely used in observational cognitive development psychology (Archer et al., 2021; Lee & Wood, 2021). Toddlers interact spontaneously with toys and screens in an unstructured environment, which allows them to observe natural behaviors in a context closer to their daily lives. A children’s carpet (120 cm long × 90 cm wide) and three toys that remained constant in all sessions (toy car, stuffed animal, and ball) were used. The dyads were asked by a female researcher to play and interact as if they were at home. A Sony HD HDR-CX160® camera was placed out of sight of the dyad. The session was recorded for 6 minutes (measured with a Model CR202 stopwatch from the Galileo Italy® line) once they were alone in the room. When the time was up, the experimenter entered and gave the pair a cell phone with a children’s video of the toddler’s preference (after asking the caregiver) and they were again asked to play and interact as if they were at home. The first and last minute and a half of the video were eliminated to avoid the fatigue and learning effect. The intermediate five minutes were then analyzed. The type of play session (toy or screen) was counterbalanced across participants, being that some participants experienced the toy session first, followed by the screen session, while others experienced the screen session first.

Two researchers analyzed the videos. Based on the interaction behaviors observed in previous studies with toddlers (Archer et al., 2021; Mundy et al., 2003), the following behaviors were coded according to established criteria: initiation of joint attention (pointing and showing an object by the toddler), responding to joint attention (following an adult’s pointing or gaze), toddlers’ verbalizations (isolated words and babble), looking at object (amount of time that the toddlers look at the screen or toy), verbal scaffolding (words directed at objects or the contents of the screen), physical scaffolding (physical guidance for the toddler to perform a task), and time offcamera. Inter-rater reliability (intraclass correlation), exceeded .85 for all measures (p < .001).

*Early Executive Functions Questionnaire Spanish Version v. 1.1.* (EEFQ, Hendry & Holmboe, 2020). A parent report scale of 28 items to measure EF between 12 and 36 months was used. Parents had to respond to a Likert scale with eight options (1. never, 2. almost never, 3. less than half the time, 4. half the time, 5. more than half the time, 6. almost always, 7. always, 8. does not apply), rating statements about their child’s behavior over the past two weeks, referring to different everyday situations where children have to regulate their behaviors, like: “Has stopped reaching for something when you have said “no/don’t touch” or something similar” or “The child has spent a lot of time trying to do something difficult”. This questionnaire has four subscales: flexibility (shifting focus to adapt to changes in the context), regulation (emotion regulation), inhibition (inhibits preponderant responses), and working memory (active manipulating of information).

This scale (Hendry & Holmboe, 2020) has three different tasks (which are then calculated with the score of each scale). They were applied by the same male researcher in a quiet room without distractions, and they were recorded. The tests were presented in order by the researcher on a table at the toddler’s height, with the primary caregiver present. Two researchers analyzed inter-rater reliability (intraclass correlation) for these tasks, and they exceeded .96 for all measures (p < .001).

- *The Waiting Game (inhibition):* The toddler is told to wait to eat an Oreo® chocolate chip cookie. The time-lapse options ranged from 0 to 30 seconds.
- *The Finding Game (working memory):* The toddler is shown how a toy was hidden in one of two opaque containers. The hiding places were interspersed four times. The number of times (from 0 to 4) that the child found the toy is counted.
- *The Sorting Game (flexibility):* five small and large spoons were given to the toddler who was asked to sort them in two different-sized transparent boxes according to the dimension (large spoons in big boxes and vice-versa). Then, they must reverse sorting (large spoons in little boxes and vice-versa). Response options ranged from not being able to sort any spoons to being able to sort them all on the reversal trial.

This scale showed adequate construct validity, limited floor and ceiling effects for subdimensions, appropriate stability, and convergent validity with parent reports of attentional control (see Hendry & Holmboe, 2020). For this sample, McDonald’s omega was from .60 to .83 for the subdimensions.

*Preschool Language Scale (Fourth Edition, PLS-5, Zimmerman et al., 2011).* To assess receptive communication skills, children were asked to point to the object corresponding to a word uttered by the experimenter. The evaluation involved determining the number of accurate identifications out of ten trials, which progressively increased in difficulty through the introduction of more distracting stimuli and challenging vocabulary. Expressive communication was assessed by promoting toddlers to verbally respond to an image presented by the experimenter, such as asking, “What is it?” The task was also video recorded. The number of correct identifications out of nine trials was recorded. A primary coder evaluated both receptive and expressive behaviors across all videos, while a second coder documented instances of these behaviors in a randomly selected subset of 15 videos (25% of the total). Inter-rater reliability (intraclass correlation) exceeded .97 for both communication measures (p < .001).

### Procedure

Free-play sessions with each dyad and cognitive tasks took place in spaces without environmental noise and adequate illumination at a daycare center at Buenos Aires from May 2022 to May 2023. To minimize potential biases in data collection, the same two researchers participated in all the evaluation and coding procedures to increase inter-rater reliability, enhances the rigor of qualitative analysis, allow data triangulation, and distribute the workload, thereby ensuring more objective and thorough results. Also, all video-recorded sessions followed a standardized protocol, ensuring consistency in instructions, environment, and materials across participants, thus reducing contextual variability. All measures were video-recorded for later analysis. At first, the toddler was placed on the caregiver’s lap or on a nearby chair, and in front of the researchers with a table in between. Then, the three tasks were applied by the same male researcher in the following order: waiting, finding, and sorting. Second, the free-play session was applied by the same female researcher. Once the behavioral evaluation was completed, both researchers read the sociodemographic and screen-use questionnaire face-to-face and they answered any questions about the research to the partens. Finally, the caregivers were given information on the healthy use of screens. The evaluation takes an hour and was performed on the same day in the morning by two psychology researchers specializing in toddlers’ cognitive development, with academic work in the area.

### Data analysis

The JAMOVI program from RStudio v. 2.4.8 was used. (The JAMOVI project, 2023). First, frequency measures of central tendency and dispersion were calculated to test the hypotheses that screen times in toddlers from 1 to 3 years old from low-to-medium SES will exceed those recommended by pediatric associations and to describe the type of content they consumed, and to describe caregivers’ perceptions on screen use. Also, the Shapiro-Wilk normality test was used, and since an abnormal distribution was found for most of the variables and due to the small sample size, non-parametric statistics were used.

The Mann-Whitney U test was used to test the hypothesis that significantly more interactions will be observed in traditional toy sessions compared to screen-based ones. Finally, Spearman correlations were applied to test whether higher screen use will be negatively correlated with cognitive skills, while screen use will be positively associated with higher SES.

The data that support the findings of this study are openly available at: https://osf.io/6rwem/?view_only=9906bbb61e464b4eb1e00c551bf0c02a

## Results

### Descriptive Statistics on Screen Use and Cognitive Variables

It was found that on average the toddlers’ reported screen use was greater than one hour for all devices, except for the PC and Tablet. The most used type of device was background TV, with a usage time of approximately five hours a day, followed by TV (approximately two hours of use) and cell phone (one hour of use). In the case of the PC and Tablet, the asymmetry values demonstrate a floor effect for the measures of the central tendency of these variables (As> 3). The results are summarized in *Table 1*.

**Table 1:** Descriptive Statistics for Screen Use and Cognitive Variables

						Asymmetry		Kurtosis	
	Mean	Med	SD	Min	Max	Asym.	EE	Kurtosis	EE
Background TV	5.300	5	4.290	0	15	.390	.40	–.864	.79
TV	2.090	1	2.078	0	7	.991	.40	–.143	.79
Cell phone	1.303	1	1.468	0	6	1.707	.40	2.970	.79
PC	.015	0	.087	0	.5	5.745	.40	33.000	.79
Tablet	.030	0	.174	0	1	5.745	.40	33.000	.79
Receptive Com.	5.000	5	4.450	0	10	.000	.41	–2.012	.80
Expressive Com.	3.656	3	3.588	0	9	.402	.41	–1.453	.80
Inhibition	36.96	36	8.545	14	50	–.342	.42	.291	.82
Flexibility	37.76	38.5	9.368	15	56	–.654	.42	.208	.83
Working Memory	37.16	39	7.710	21	49	–.559	.42	–.375	.83
Regulation	38.48	40	11.65	9	55	–.583	.40	–.186	.79

The types of content entertainment (*n* = 13, 39.4%), educational (*n* = 10, 3.3%), and music (*n* = 10, 3.3%) were distributed almost equally in the sample, while the majority of toddlers consumed content appropriate for their age (*n* = 25, 75.8%) and the minority consumed content for adults and/or older children (*n* = 8, 24.2%).

### Open Responses on Screen Use

For the effect that caregivers think screens have on their toddlers, two (6%) stated that they did not know the effects, 18 (50%) that they have negative effects (e.g., “It hurts the eyes,” “They lose concentration.”, “They are addictive”, “They cause tantrums”), six (16.6%) that they have mixed effects (e.g., “It is positive, because it teaches him colors, numbers, animals”, “With the screens he stops socializing, does not enjoy the surroundings, withdraws”, “It is bad for the eyes, but that can be avoided with responsible use”), and eight (22.2%) only stated positive effects (“They can learn things from screens, like animals. We also use it to dance”. “Teaches”).

Regarding whether caregivers prefer traditional toys or screens, 28 primary caregivers (84.8%) responded that traditional toys are more favorable than screens (e.g., “Toys are better because screens can expose children to content that may not be appropriate for their age“, “With toys you can give them things appropriate for their age”, “Toys because they learn to grasp more, they know how to move, they know what they are doing”, “Toys, imagination”), while five (8.44%) responded that both are necessary for the education of their toddlers (“Both, because the screen has many different animals, and with toys, you cannot buy everything they see on the screens”, “Screens, he knows things through screens”, “Both, because screens can teach and so can toys. Combining makes it possible to identify what is used on the screen”. No primary caregiver stated that they prefer screens to traditional toys.

Regarding whether they know the recommendations of pediatric societies, 27 (81.1%) of the adults stated that they did not know them, and only 6 (18.9%) said that they knew them (*e.g*., “Yes, it is harmful to the eyes”, “Yes, the child should watch a maximum of one hour per day”, “My private pediatrician says that we should reduce the screen time due to visual problems and sedentary lifestyle”).

Finally, regarding why they are given screens, 30 (9.1%) caregivers responded that they used them to distract their toddlers while they were doing something else, to keep them calm or so that they could do another activity (*e.g*., “Because I have to go to work and get distracted for a moment. So that she doesn’t cry”, “So that she stays calm, we give her the phone so she can calm down”) while only 3 (9.9%) stated that they never leave their toddler alone with the screen (“She is never alone with screens. At most if we go in the car”, “I don’t give them to him, I leave him toys, markers and little books”).

### Comparison of Communication Behaviors Based on Free Play with a Toy or Screen

Regarding communication behaviors of the dyads, it was found that in most cases both the caregiver’s and the toddler’s behaviors were significantly greater in terms of communication during the free-play session with traditional toys than with screens. Specifically, more response behaviors and initiation of joint attention and toddlers’ verbalizations were found in the test with traditional toys, with moderate to high effect sizes for this age range. The same results were found with maternal verbal and physical scaffolding behaviors. The results are summarized in *Table 2*.

**Table 2:** EF and Early Communication by Type of Objects during Free-Play Session

	Type of Free-Play Session							
	Traditional Toys			Cell Phone			U	Rosenthal
	Range	MR	SR	Range	MR	SR		
**Toddler Interaction Behaviors**								
Responding to joint attention	0–	16 42.95	1374.5	0–5	22.05	705.5	177.5	.639^***^
Initiation of joint attention	0–	11 34.95	1118.5	0–10	28.95	897.5	401.5	.203
Initiation of behavioral request	0–	10 39.80	1273.5	0–3	25.2	806.5	278.5	.505^***^
Verbalizations	0–	15 41.06	1314	0–6	23.94	766	238	.496^***^
**Adults Interaction Behaviors**								
Verbal scaffolding	0–	28 41.37	1282.5	0–17	22.92	733.5	205.5	.561^***^
Physical scaffolding	0–	14 43.16	1381	0–7	21.84	699	171	.691^***^
Offcamera (seconds)	0–	240 34.61	1073	0–264	29.47	943	415	.148

*Notes: MR: Mean Ranks, SR: Sum of Ranks.*

### Correlations Between Interaction Variables During Free-Play Sessions and Cognitive and Screen Use

In free-play sessions with traditional toys and cell phones, positive correlations between interaction behaviors with communication and executive functions variables were found. Specifically, there were positive associations between the initiation of joint attention and toddlers’ verbalizations with receptive and expressive communication, working memory, and cognitive flexibility. The effect size was generally higher in traditional toy free-play sessions than in cell phone play sessions.

Regarding screens, only background TV and TV use were positively associated with responding to joint attention during cell phone free sessions, and to initiation of joint attention in free-play sessions, and there was a negative association with TV use and toddlers’ verbalization during traditional free-play sessions with toys. These results are shown in *Tables 3* and *4*.

**Table 3:** Associations Between Communication, EF, and Sociodemographic Variables with Interactions During Traditional Free-Play Session with Toys

	Communication		Executive Functions				Screen Use Reports				
	Receptive Communication	Expressive Communication	Cognitive Flexibility	Inhibition	Working Memory	Regulation	Background TV	TV	PC	Cell Phone	Tablet
**Toddler Interaction Behaviors**											
Responding to joint attention	-.300	-.267	-.298	-.230	.054	.054	.410*	.439**	-.334	-.038	-.061
Initiation of joint attention	.571**	.306	.313	.340	.436**	.128	-.115	-.100	-.190	-.013	.064
Initiation of behavioral request	.400*	.203	.247	.291	.355	.286	-.224	-.178	-.218	-.237	-.073
Verbalizations	.724***	.555**	.525**	.534**	.495**	.152	-.284	-.408*	-.295	-.036	-.057
**Adults’ Interaction Behaviors**											
Verbal scaffolding	.285	.140	.187	.213	.489**	.046	-.048	-.133	-.178	.007	.175
Physical scaffolding	.272	.182	.135	.154	.311	.202	-.262	-.176	-.207	-.062	.065
Off camera	-.176	-.052	-.010	-.228	-.301	-.468	-.019	.148	.300	.081	-.264

*Note: Spearman Rho partial correlation. Inserting age as a covariable.*

**Table 4:** Associations Between Communication, EF, and Sociodemographic Variables with Interactions During Cell Phone Free-Play Session

	Communication		Executive Functions				Screen Use Reports				
	Receptive Communication	Expressive Communication	Cognitive Flexibility	Inhibition	Working Memory	Regulation	Background TV	TV	PC	Cell Phone	Tablet
**Toddler Interaction Behaviors**											
Responding to joint attention	-.085	.014	-.057	-.024	.040	.198	.125	.212	-.103	.162	-.069
Initiation of joint attention	.126	.103	.302	.283	.432*	.134	.240	.364*	-.174	-.292	-.088
Initiation of behavioral request	-.058	-.049	-.018	-.041	-.065	.285	-.130	-.057	-.109	-.261	-.204
Verbalizations	.568**	.357	.425*	.366	.235	.253	-.127	-.161	-.220	.061	-.158
**Adults Interaction Behaviors**											
Verbal scaffolding	.315	.189	.093	.185	.436*	.067	.006	-.062	-.290	.235	.218
Physical scaffolding	.208	.345	.327	.057	.455*	-.166	-.166	.017	-.201	.022	.192
Off camera	.189	.337	.054	-.102	-.116	-.276	-.276	-.064	-.140	.027	-.140

### Correlation Between Screen Use, Cognitive and Sociodemographic Variables

Regarding the use of screens and cognitive variables, only statistically significant and negative associations were found between the time spent with background TV and receptive (*Rho* = -.463, *p* = .008), expressive communication (*Rho* = -.533, *p* = .002), and cognitive flexibility (*Rho* = -.492, *p* = .006). Longer caregivers reported that the toddler’s time exposed to background TV was related to lower performance on cognitive variables. Finally, more UBNs were related to higher TV use (*Rho* = .578, *p*< .001). No significant associations were found between the other types of screens and the communication and regulation variables (p> .05). The results of the correlations are presented in Table 5.

**Table 5:** Correlations Between Communication, Executive Function, Screen Use, and Sociodemographic Variables

	Receptive Com.	Expressive Com.	Inhibition	Flexibility	WM	Regulation	Back-ground TV	TV	Tablet	PC	Cell phone	UBN
Receptive Com.	-											
Expressive Com.	.788 ***	-										
Inhibition	.543 **	.389 *	-									
Flexibility	.720 ***	.841 ***	.564 **	-								
WM	.546 **	.475 **	.414 *	.582 ***	-							
Regulation	.139	.274	.059	.376 *	.240	-						
Background TV	-.463 **	-.533 **	-.077	-.492 **	-.182	-.169	-					
TV	-.113	-.218	-.009	-.273	.005	-.270	.578	-				
Tablet	-.109	-.209	-.246	-.279	-.097	-.046	-.009	-.066				
PC	-.238	-.070	-.020	-.054	-.065	-.223	.056	-.066				
Cell phone	.129	-.023	.209	-.134	.105	-.233	.233	.151				
Amount UBN	-.067	-.052	-.009	-.141	.088	-.149	.233	.434				

*Note: Com = Communication, WM = Working Memory, UBN = Unsatis! ed Basic Needs.*

## Discussion

The objective of this research was to describe the use of screens in a non-WEIRD sample from Buenos Aires, to explore parents’ perception of screens effects on toddlers’ development, compare interactions between the primary caregiver and toddler dyads considering the type of objects they used to play (cell phones or traditional toys), and associate these communicative interactions with toddlers’ cognitive ability, screen times reported by caregivers, and sociodemographic variables. Regarding the hypothesis that screen times in 1-to-3-year-old toddlers from low-to-medium-SES will exceed that recommended by pediatric associations, it was found that screen times were longer than recommended by these associations and that the content consumed was diverse. Most caregivers believed that screens produced negative effects, and that it is better to use traditional toys. They reported not knowing the recommendations of pediatric associations and used screens as a distraction for their toddlers.

About the first research question, differences were found when observing the dyads’ verbal and no-verbal interactions depending on which mediating object they used for 5-minute free-play sessions. A greater number of verbal and non-verbal interactions were found for both toddlers and caregivers in the free-play sessions with traditional toys, and in both sessions, corroborating the hypothesis, and the interaction behaviors were positively associated with the cognitive skills evaluated.

Considering the second research question about how a caregiver’s report of toddler’s screen time use with different devices related to communication and regulation skills, considering sociodemographic variables in a non-WEIRD sample, the hypothesis was partially corroborated. TV and background TV use were positively associated with a toddler’s nonverbal interaction behaviors and parents educational level, and negatively associated with the number of unmet basic needs. Finally, toddlers’ verbal interactions were negatively associated with TV use only in the free-play session with toys.

As in other empirical and review studies (Gago Galvagno et al., 2022a; Madigan et al., 2020; Medawar et al., 2023; Panjeti-Madan et al., 2023; Rayce et al., 2024) screen time was greater than recommended by pediatric associations. These antecedents also coincide with the results of this research in that, on average, children were exposed to more than one hour per day, specifically to cell phones, TV, and background TV.

About the associations between communication, regulation, and screen use, the results also coincide with previous research (Cliffet al., 2018; Gago Galvagno et al., 2020, Madigan et al. 2021; Lee et al., 2024), since the greater the screen times, the lower the reported regulation and language scores. However, it is necessary to highlight that only background TV effects were significant. This could be because that is what toddlers are exposed to for the longest time, and as some studies show, such exposure hinders communication channels at home and generates distracting stimuli in the environment that could reduce toddlers’ sustained attention (Nichols, 2022; Ribner et al., 2021). Furthermore, caregivers’ responses in the open question section indicated that they generally use screens to distract their toddlers. Therefore, it could be inferred that they do not usually accompany this activity. The absence of associations with other types of screens could be due to low time of use, the accompaniment of caregivers during their use, or the fact that toddlers mostly see content appropriate for their age.

On the other hand, the cognitive regulation and communication skills measured with the behavioral tests were positively associated with the behaviors performed by both the toddler and the caregiver during both sessions. Toddlers with higher and more regulated levels of communication could be more receptive and elicit more behaviors, which would produce more initiations and responses of joint attention and verbalizations in parents. These results coincide with those of Archer et al. (2021), who compared children’s use of their own technological devices with novel ones, and those of Lee and Wood (2021) with objects in 2 and 3 dimensions, and highlight that the cognitive abilities of toddlers are important when considering the type of interactions that they carry out during interaction spaces, regardless of the type of mediating object that was used. This highlights the importance of interventions that also consider toddlers’ cognitive abilities and optimizing how parents engage with their children’s interactions.

Regarding group comparisons, the greater number of interactions during free-play sessions with toys compared to screen sessions is congruent to previous work in many senses and to Sociocultural Psychology theory. First, studies have shown a negative correlation between screen use and these abilities, so this lower amount of communication and regulation (cognitive flexibility) could be partly because the screen did not elicit verbal behaviors or active interactions, distracts attention from another stimulus and the environment (so there is not a constant shifting attention, fundamental to cognitive flexibility), and is, in general, a passive activity (Gago Galvagno et al., 2022b; Madigan et al., 2021), as also demonstrated by the qualitative results of this study. Toys are mediating objects that could promote cognitive development. The lack of associations with inhibition, working memory, and regulation could be because background TV contributions primarily affect areas of cognitive development that are more directly related to social interaction and active learning, such as cognitive flexibility.

Second, and in line with the previous paragraph, sharing toys with toddlers involves the use of stories, labeling objects verbally, moving them closer and further away from the toddler, and producing sounds so that the toddler can interact with them (Sosa, 2016). On the other hand, screen sharing involves showing the toddler certain content, maintaining a stable posture, and pointing to and naming characters or situations that occur. Therefore, it could be stated that the two situations generate interaction dynamics that are very different from each other, with toys promoting and requiring verbal and physical interactions to generate a communicative environment, while the use of screens can dispense with any type of communication to be attractive to toddlers.

Lastly, the small number of interactions that adults carry out with toddlers during interacting sessions with screens is very striking. This could be because toddlers spend more time on screens alone compared to sharing screens with an adult. As caregivers stated, using screens to distract the children means that they are alone during use. It would be important at this point to promote caregiver communication while using screens with the toddler so that they can generate greater pointing and verbalizations during these periods, and thus contribute to the toddler’s cognitive development. Previous research shows that the more scaffolding on the part of the caregiver during the use of screens, the fewer the negative effects generated by them (Gago Galvagno, et al., 2022b; Karani et al., 2022; Li et al., 2020).

Regarding the differences in the associations between screen use, verbal and non-verbal communication, it could be interpreted that the positive associations of non-verbal behaviors (joint attention and responding to joint attention) are due to the higher consolidation of these behaviors within the study age range, which makes them more readily available for toddlers to use them during interactions (Miller & Marcovitch, 2015; Simaes et al., 2022). Additionally, screens could elicit more non-verbal behaviors because the toddler can point out and share with his or her caregiver the content that is being viewed. Regarding the negative associations of verbal communication and TV use, they go hand in hand with other studies (Li et al., 2020, Madigan, 2021; Rayce et al., 2024). It could be expected that, during free-play sessions with toys, toddlers who are more accustomed to watching TV interact less verbally when using toys because: a) they are less accustomed to traditional games; b) they already have a lower verbal repertoire due to the time spent with screens; c) caregivers do not know how to play during these sessions, and therefore elicit the toddler’s verbal behavior to a lesser extent.

## Conclusion

The relevance of this study lies in the fact that it works with a non-WEIRD sample of dyads: Argentine toddlers and caregivers of low-to-medium SES. Most studies have used questionnaires and measured verbal communication variables, whereas this study is behavioral and measures both verbal and non-verbal communication and regulation variables. The results of the study highlight the importance of continuing to teach responsible use of screens, promoting free play with toys, working with the type of interactions that caregivers engage in during play with screens, and highlighting the possible negative effects of screens on verbal communication and regulation. It would be beneficial to explore strategies to balance screen times with activities that encourage face-to-face communication and social interaction, thereby strengthening communication and emotional regulation skills in children and adults.

## Limitations

The sample size was small and obtained with non-probabilistic sampling. The design was transversal and correlational; therefore, causality cannot be established or the development between the variables observed. Also, although communication and regulation were observed behaviorally, the time spent using screens at home was reported by the caregivers and it was not measured whether they shared their use at home.

Future studies would benefit from using larger sample sizes and probabilistic sampling. In addition, a longitudinal study could be carried out to evaluate how interactions vary over time for different play sessions. Finally, a more ecological evaluation of screen use could also be carried out at home itself where interactions occur. This would pave the way for intervention studies, aimed to promote adequate knowledge and responsible screen use practices to protect and stimulate toddlers’ development.
